# A reliability study of the rapid emergency triage and treatment system for children

**DOI:** 10.1186/s13049-016-0207-6

**Published:** 2016-02-24

**Authors:** Brita Henning, Stian Lydersen, Henrik Døllner

**Affiliations:** Department of Pediatrics, St. Olav’s University Hospital, Trondheim, Norway; Regional Centre for Child and Youth Mental Health and Child Welfare, Trondheim, Norway; Department of Laboratory Medicine, Children’s and Women’s Health, Norwegian University of Science and Technology, Trondheim, Norway

**Keywords:** Triage, RETTS-p, Reliability, Pediatric emergency care

## Abstract

**Background:**

To evaluate inter- and intrarater reliability of a new Scandinavian triage system for children, the Rapid Emergency Triage and Treatment System-pediatric (RETTS-p).

**Methods:**

Two observational studies were conducted at the Pediatric Emergency Department (PED), St. Olav’s University Hospital, Trondheim, Norway. Using RETTS-p, nurses assign one of five triage priority levels to each patient on the basis of clinical signs and symptoms evaluations and vital parameter measurements.

*Study 1:* Prior to the introduction of RETTS-p in 2012, all nurses in the PED completed a theoretical and practical training. Four months later, 19 nurses triaged 20 fictive but realistic pediatric cases two times 9 months apart (Waves A and B). *Study 2:* Nurse pairs consisting of a regular nurse and a research nurse simultaneously and independently triaged 200 pediatric patients who were referred with various common medical and surgical complaints.

**Results:**

*Study 1:* Kendall’s W for Waves A and B were 0.822 and 0.844, respectively. Using a mixed linear model, we found no difference in triage priority levels between Waves A and B. Compared to a consensus level made by the research group, the nurses rated 85.1 % fictive cases correctly, and 99 % were rated correctly or within one adjacent priority score. *Study 2:* The interrater correlation coefficient in a linear mixed model was 0.762, confirming a high interrater reliability in real-life triaging.

**Discussion:**

We found a very high degree of agreement between nurses who used RETTS-p to prioritize children, both in a theoretical case scenarios study, but also in real-life triaging.

**Conclusions:**

RETTS-p may be a credible and robust triage system, but it has not been validated yet.

**Electronic supplementary material:**

The online version of this article (doi:10.1186/s13049-016-0207-6) contains supplementary material, which is available to authorized users.

## Background

In hospital emergency departments (ED), it is important to prioritize patients to ensure that the sickest patients are evaluated and treated first. To undertake effective prioritization, several triage systems have been developed for adults and children [1–7]. The Rapid Emergency Triage and Treatment System, RETTS, previously called METTS, was developed at Sahlgrenska University Hospital, Sweden, and it has been increasingly used in Sweden and other Scandinavian countries [8–10]. An English online version has been developed, making the system internationally available, in line with more established triage systems [11]. Triage with RETTS may be advantageous because it is based both on vital parameter (VP) measurements and evaluation of individual disease manifestations. The VP priority levels in the pediatric version (RETTS-p) are age adjusted. They originally were based on Canadian experience [12] and were adjusted later [13]. RETTS-p includes more than 100 common pediatric disease manifestations, which are categorized in 40 Emergency Signs and Symptoms (ESS) algorithms [11]. Both VPs and ESSs are scored in one of five priority levels by ED nurses, and the final triage priority rating is determined as the highest of the VP and ESS scores [11]. RETTS-adults has been shown to be a valid and reliable system [8], but the validity of the pediatric version has not been studied yet. Recently, a good to very good reliability was reported when Swedish nurses from a mixed adult and pediatric ED triaged pediatric case scenarios [14].

Norwegian versions of RETTS were implemented at the ED, St. Olav’s University Hospital, Trondheim, Norway, in 2011, and as the first hospital in Norway, we introduced an adapted Norwegian RETTS-p in the PED in 2012. We now aim to study the reliability (inter- and intrarater agreement) of the Norwegian RETTS-p as used by nurses in a large Norwegian PED.

## Methods

Two observational studies were performed at the PED, Department of Pediatrics, St. Olav’s University Hospital, Trondheim, Norway, during the period from April 2013 to February 2014. The Department of Pediatrics provides emergency care for a population of approximately 58,000 children aged < 16 years, of whom 18,000 are less than 5 years of age (Statistics Norway, 2014). In 2013 the PED received 4223 children aged 0–16 with various pediatric (*n* = 3167, 75 %), surgical (*n* = 506, 12 %), neurosurgical (*n* = 211, 5 %), orthopedic (*n* = 106, 2.5 %), and other complaints (*n* = 233, 5.5 %). The largest patient group was children with respiratory tract infections (*n* = 699, 17 %). Children with life-threatening illness, including those with multitrauma, usually were not received in the PED but in the ED for adults and are not included in the studies. Nearly all children were referred after being assessed in the primary care system by general practitioners or at the primary care ED. In the PED more than half of the patients were treated as outpatients, and approximately 40 % were admitted to the pediatric wards. In 2013, RETTS-p triage priority ratings in the entire PED population were: red 8.7 % of the admitted children, orange 30.3 %, yellow 29.5 %, green 18.8 % and blue 12.7 %.

### Description of the rapid emergency triage and treatment system for children

Triage with RETTS-p is based on a combination of VP measurements and evaluation of individual disease manifestations (Emergency Signs and Symptoms, ESS) (Additional files 1 and 2) [8, 11]. The VPs include airway, respiratory rate, oxygen saturation, heart rate, alertness level as measured by the Glasgow Coma Scale, and temperature, and the VP priority levels are age adjusted. Forty ESS algorithms each cover one or more of more than 100 common acute pediatric, surgical, and orthopedic complaints, e.g., respiratory difficulty and apnea, which are included in ESS no. 104, abdominal pain, constipation, and diarrhea (ESS no. 106), head trauma (ESS no. 130), and uncomfortable parents (ESS no. 153), respectively. Both VPs and ESSs are scored in one of five priority levels by ED nurses. Priority level red is defined as urgent need of medical doctor examination, orange implies medical doctor examination no later than 20 min, triage priority rating yellow means that the patient should be evaluated within no more than 120 min, and green demands examination before 4 h. Blue level indicates no need for triage and examination at the ED, and this patient group was not included in the present studies. The final triage priority rating is determined as the highest level from the VP and ESS ratings. Each ESS algorithm in addition includes recommendations for initial basic evaluation and treatment, such as blood sugar level testing and oxygen treatment. In the present studies, we used a Norwegian version of the RETTS-p (version 1.2), which was translated and adapted to Norwegian conditions by the research group.

### Study design

#### Study 1

Prior to the introduction of RETTS-p in 2012, all nurses in the PED completed a theoretical and practical training. Four months later, 19 nurses triaged 20 written but realistic patient cases (Additional file 1), which were based on representative real-life referrals to our PED (Wave A). Nine months later, 12 nurses who were still working in the PED triaged the same cases again (Wave B).

#### Study 2

Pairs comprising one out of 20 regular nurses and one out of four research nurses simultaneously and independently triaged 200 children who were referred with either pediatric (*n* = 150), surgical (*n* = 30), neurosurgical (*n* = 10) or orthopedic (*n* = 10) complaints during the time period from June 2013 to February 2014. The children were included when one of the research nurses was available on dayshifts and afternoon shifts. The distribution of the included children was quite similar to the distribution of children in the entire PED population: pediatric patients 75 vs 75 %, surgical patients 15 vs 12 %, neurosurgical patients 5 vs 5 % and orthopedic patients 5 vs 3 %. In the emergency room, the regular nurse triaged the patients as usual with written documentation of VPs, ESS number, and triage priority scores. Simultaneously, the research nurse as a silent observer received all objective measurements, apart from evaluation of alertness and respiratory rate, which were observed by the research nurse herself, and independently triaged the patient. Usually, the research nurses worked in the PED and had more experience in pediatric nursing care compared with the regular nurses (mean 17 vs. 5 years). Also, the proportions with continuing education in nursing were higher (100 vs. 40 %, respectively).

### Ethics

The study was approved by the institutional review board at St. Olav’s University Hospital and the Regional Ethics Committee, Mid-Norway. All nurses in our emergency department were asked to participate (orally). Written consent to participation in Study 1 was documented. In Study 2 the research nurse prior to triaging informed the regular nurse, the child and the caregiver, and oral consent to participation was collected from the regular nurses. The hospital review board approved this approach.

### Statistical analysis

In Study 1, the inter-rater agreement was measured in Waves A and B separately using Kendall’s W coefficient of concordance [15], which has a similar interpretation as a correlation coefficient. In Wave A, 13 single values were missing for six nurses. That is, 13 values were missing out of 19 × 20 = 380 values (3.4 %). In Wave B, 4 single values were missing for four nurses. That is, 4 values were missing out of 12 × 20 = 240 values (1.7 %). The missing values were singly imputed using the expectation-maximization algorithm, making it possible to use all 19 (12) nurses in the analysis. We used a linear mixed effects model to estimate the variance components due to variance between patient cases and variance between raters, as well as to study intrarater agreement (i.e., whether there was a “learning effect” from Wave A to Wave B). The triage priority scores made by the nurses were furthermore compared with consensus triage priority scores as determined by the research group.

In Study 2, data were missing on 20 patients: Triage priority ratings were not available for the regular nurse in 5 patients and for the research nurse for 15 patients. Hence, 180 patient cases were available for analysis, including 20 regular nurses and four research nurses. We used a linear mixed model with triage priority score (Red = 1, Orange = 2, Yellow = 3, Green = 4) as dependent variable, patient and nurse as crossed random factors, and a fixed effect of research nurse (vs. regular nurse). The interrater reliability measured as the intraclass correlation coefficient (ICC) in this model was estimated as [16].

$$ \widehat{ICC}=\frac{Variance(patients)}{Variance(patients)+ Variance(raters)+ Residual}. $$

This ICC is equivalent to Cohen’s quadratic weighted kappa [17], as also noted by van Veen et al. [7].

The ICC was calculated using Stata 11, and the other analyses were done in SPSS 22.

## Results

### Study 1

#### Agreement among nurses triaging written patient cases

Agreement in the total priority triage score between nurses who triaged 20 written realistic patient cases in Study 1 was measured separately in Wave A and Wave B. Kendall’s W for Waves A and B were 0.822 and 0.844, respectively. The data included all 603 observations when 19 nurses in the first wave and 12 nurses in the second wave independently triaged the written cases. In a linear mixed effect model including a fixed effect of Wave B, the estimated average rating at Wave A was 2.148, and the average rating at Wave B was 0.0439 (*p* = 0.168) higher. Since this is far from significant, we removed wave from the model. The average score of the reduced model was 2.208, and the total variance was 0.769 = 0.877^2^, including the variance between the rated patients (0.627 = 0.792^2^), plus the variance due to the raters (0.00212 = 0.046^2^) and the residual variance (0.139 = 0.373^2^). The interrater reliability (ICC) estimate was 0.816.

#### Proportion of the ratings giving the “correct” triage rating

Among the 603 ratings in total in Waves A and B, 513 (85.1 %) gave the “correct” triage level compared to consensus, 597 (99 %) were either correct or within one adjacent triage priority level, and 6 ratings were two priority levels higher than consensus (Tables 1, 2 and 3).

**Table 1:** Nurses’ triage priority ratings compared to consensus priority ratings^a^

Nurse triage priority ratings	Consensus triage priority ratings				
	Red	Orange	Yellow	Green	Total
Red	88	36	0	0	124
Orange	2	249	19	6	276
Yellow	0	15	129	9	153
Green	0	0	3	47	50
Total	90	300	151	62	603

^a^In Study 1, Wave A (*n* = 367 ratings) and Wave B (*n* = 236 ratings) combined

**Table 2:** Nurses’ triage priority ratings of 20 fictive cases^a^

Fictive patient case number	Nurses triage priority ratings				
	Red	Orange	Yellow	Green	Total ratings
	N (%)	N (%)	N (%)	N (%)	N
1	1 (3)	29 (94)^b^	1 (3)	0	31
2	12 (40)	18 (60)^b^	0	0	30
3	0	0	3 (10)	28 (90)^b^	30
4	29 (97)^b^	1 (3)	0	0	30
5	10 (32)	19 (62)^b^	2 (6)	0	31
6	5 (17)	24 (83)^b^	0	0	29
7	2 (6)	29 (94)^b^	0	0	31
8	0	27 (90)^b^	3 (10)	0	30
9	30 (100)^b^	0	0	0	30
10	0	1 (3)	29 (94)^b^	1 (3)	31
11	1 (3)	26 (90)^b^	2 (7)	0	29
12	0	22 (76)^b^	7 (24)	0	29
13	0	11 (37)	19 (63)^b^	0	30
14	1 (3)	28 (97)^b^	0	0	29
15	0	2 (7)	28 (93)^b^	0	30
16	0	0	29 (94)^b^	2 (6)	31
17	0	6 (19)	6 (19)	19 (62)^b^	31
18	4 (13)	27 (87)^b^	0	0	31
19	0	5 (17)	24 (83)^b^	0	29
20	29 (97)^b^	1 (3)	0	0	30
Total	124 (21)	276 (46)	153 (25)	50 (8)	603

^a^In Study 1, Wave A (*n* = 367 ratings) and Wave B (*n* = 236 ratings) combined

^b^“Correct” priority rating as determined by the research group

**Table 3:** Correct and incorrect triage priority ratings of each Nurse^a^

Nurse	Consensus ratings^b^	Non-consensus ratings	Total ratings
	N (%)	N (%)	N
1	17 (85)	3 (15)	20
2	17 (85)	3 (15)	20
3	33 (83)	7 (17)	40
4	26 (67)	13 (33)	39
5	36 (92)	3 (8)	39
6	34 (87)	5 (13)	39
7	17 (85)	3 (15)	20
8	28 (72)	11 (28)	39
9	14 (78)	4 (22)	18
10	33 (83)	7 (17)	40
11	37 (95)	2 (5)	39
12	32 (94)	2 (6)	34
13	18 (100)	0	18
14	38 (95)	2 (5)	40
15	35 (88)	5 (12)	40
16	18 (90)	2 (10)	20
17	39 (98)	1 (2)	40
18	27 (69)	12 (31)	39
19	14 (74)	5 (26)	19
Total	513 (85.1)	90 (14.9)	603

^a^In Study 1, Wave A (*n* = 367 ratings) and Wave B (*n* = 236 ratings) combined

^b^As determined by the research group

The estimated coefficient based on a logistic mixed model including a fixed effect of Wave B was 2.015 for Wave A and 1.862 (*p* = 0.58) for Wave B. After removing the nonsignificant effect of wave from the model, the resulting model gave these estimates (on a log odds scale): variance between patients 0.9988 (*p* < 0.001) and variance between nurses 0.5272 (*p* < 0.001). Hence, we see that the probability of giving the correct priority rating varied significantly between patient cases and between nurses, and among these, the variation between patient cases was largest. Indeed, only patient case no. 9 was triaged correctly by all nurses (Table 2). In case no. 2, only 18 out of 30 nurse evaluations (60 %) were equal to the consensus level and 12 evaluations were one level higher than consensus (Table 2). In case no. 17, 6 nurses rated one priority level, and 6 nurses rated two priority levels higher than consensus (Table 2). Taken together, overtriage appeared in 63 (11.6 %) evaluations among which 57 were one level and 6 were two levels higher than consensus, and undertriage appeared in 20 evaluations (3.3 %), all of which were one level lower than consensus (Tables 1 and 2). Regarding the nurses, 1 rated all, 13 rated 83 to 98 %, and 5 nurses rated only 67 to 78 % of the cases correctly (Table 3).

### Study 2

#### Referred pediatric patients: agreement between regular nurses and research nurses

Two hundred patients admitted to the PED were included in Study 2. The 20 regular nurses each rated on average 9 patients (median 4, range 1 to 30), and the four research nurses rated 25, 44, 55, and 56 patients, respectively.

The results of the ratings are shown in Table 4. The estimated variance components in the model were variance in between the rated patients (0.5185 = 0.7201^2^), variance due to raters (4.13*10^−18^ = (2.03*10^−9^)^2^), and the residual variance (0.1683 = 0.4102^2^). The interrater reliability (ICC) estimate was 0.762. The research nurses rated 0.0889 (95 % CI 0.0042 to 0.1736, *p* = 0.040) higher than regular nurses; that is, they tended to give lower priority to the patients. A closer look at the distributions for regular nurses versus research nurses in Table 4 reveals that the ratings varied at several levels, and the largest difference between the two was for priority “green”: The regular nurses and the research nurses rated 27 and 40 of the 180 patients, respectively as “green”.

**Table 4:** Final triage priority ratings comparing 20 regular nurses and 4 research nurses^a^

Regular nurse triage priority ratings	Research nurse triage priority ratings				
	Red	Orange	Yellow	Green	Total
Red	6	5	1	0	12
Orange	1	55	4	3	63
Yellow	1	8	52	17	78
Green	0	0	7	20	27
Total	8	68	64	40	180

^a^In Study 2 (*n* = 180 observations)

### Final RETTS-p priority rating versus priority levels from VP measurements and ESS algorithms

The final RETTS-p triage priority rating is determined as the highest of the priority levels of the VP measurements and the chosen ESS algorithm. Out of 595 ratings in the first study, VP and ESS rated equally in 279 (46.9 %); in 96 (16.1 %) ratings, VP priority levels were higher than ESS, whereas in 220 (37.0 %), the ESS priority levels were highest (Table 5). In Study 2, among 176 ratings performed by the regular nurses, VP and ESS rated equally in 67 (38.1 %); VP was highest in 60 (34.1 %) ratings, and ESS was highest in 49 (27.8 %) (Table 6). Among 180 research nurse ratings, the corresponding figures were 84 (46.7 %), 55 (30.6 %), and 41 (22.7 %) (Table 7).

**Table 5:** Final triage by Vital Parameters (VP) and Emergency Signs and Symptoms (ESS) algorithms priority levels^a^

VP triage priority levels	ESS algorithms triage priority levels				
	Red	Orange	Yellow	Green	Total
Red	10	3	0	1	14
Orange	53	137	30	12	232
Yellow	51	50	75	50	226
Green	1	39	26	57	123
Total	115	229	131	120	595^b^

^a^In Study 1 performed by 19 nurses in Wave A (*n* = 367 ratings) and 12 nurses in Wave B (*n* = 236 ratings)

^b^8 VP or ESS ratings were missing

**Table 6:** Final triage by Vital Parameters (VP) and Emergency Signs and Symptoms (ESS) algorithms priority levels (Regular nurses)^a^

VP triage priority levels	ESS algorithms triage priority levels				
	Red	Orange	Yellow	Green	Total
Red	2	4	1	0	7
Orange	2	16	16	11	45
Yellow	1	11	25	28	65
Green	0	9	26	24	59
Total	5	40	68	63	176^b^

^a^In 180 ratings in Study 2 performed by regular nurses

^b^4 VP or ESS ratings were missing

**Table 7:** Final triage by Vital Parameters (VP) and Emergency Signs and Symptoms (ESS) algorithms priority levels (Research nurses)^a^

VP triage priority levels	ESS algorithms triage priority levels				
	Red	Orange	Yellow	Green	Total
Red	1	2	2	0	5
Orange	1	25	11	14	51
Yellow	1	11	17	26	55
Green	0	7	21	41	69
Total	3	45	51	81	180

^a^In 180 ratings in Study 2 performed by research nurses

## Discussion

We found a very high inter- and intrarater reliability of the Scandinavian triage system for children RETTS-p; i.e., there was a high degree of agreement between nurses when they evaluated how fast referred children should be seen by a medical doctor. The nurses rated 85 % written, realistic pediatric cases at the correct priority level, and nearly all (99 %) were rated correctly or within one adjacent level. Undertriage one level appeared in only 3.3 % of cases and three times less frequently than overtriage, which is important from the perspective of identifying urgent patients. The findings from the theoretical study with case scenarios were largely confirmed in real-life triaging of children who were referred with common pediatric and surgical complaints. Our findings are in line with data from a recent Swedish study where nurses in a mixed adult-pediatric ED in a general hospital triaged 40 fictive pediatric cases, but it is reassuring that we now have confirmed the repeatability of RETTS-p in real-life triage in a PED.

Our PED population includes relatively few children with life-threatening illness, and because in Norway nearly all cases are referred after evaluation in the primary care system, we did not receive many with mild diseases. These patient groups may be the most easy to triage. Therefore, it is noteworthy that in the two present studies, the variances in triage priority ratings between patients were larger than the variances between raters (nurses). Some patients just seem to be more difficult to triage than others, independent of the nurse’s experience. On the other hand, in the fictive case study, some nurses triaged more precisely than others, and the real-life data showed that the research nurses, who were more experienced, had a tendency to triage many patients at a lower level. Hence, less experience may explain at least some of the variation in rating between nurses.

All PED nurses were trained practically and theoretically in RETTS-p prior to the implementation of the system, and in Study 2 new nurses who started during the study period received the same training. In both studies we found a very high interrater agreement, and through testing two times in the fictive case study, we also found a high intrarater agreement but no learning effect during a 9-month period when the nurses gained more experience with the system. From these findings, we suggest that RETTS-p may be a credible and robust triage system that nurses quickly learn to manage.

Currently, no data support using one pediatric triage system instead of another [5], but test performances in the present studies were not lower than in studies of other pediatric triage systems [5]. RETTS-p was developed for Scandinavian countries, but available English versions will enable use abroad. Triage in RETTS-p uses both VP measurements and clinical signs and symptoms to prioritize children. In the present studies, we found that the final triage priority rating was based on similar VP and ESS priority levels in approximately half of the cases, whereas in the rest, the final triage priority rating was due to either the priority levels of the VP or the ESS, which may support the superiority of including both physiological measurements as well as clinical manifestations in the assessments. However, urgency in the need of help does not always equals disease severity, and therefore the evaluation of RETTS-p should be complemented with studies on the validity; i.e., it should be studied if triage priority ratings in RETTS-p are associated with markers of disease severity in PEDs.

There may be some limitations of the study. Because in Norway most children are referred to hospital PEDs after initial evaluation in the primary care system, the study populations were selected, including a few with mild conditions. In addition, only a few with life-threatening conditions were included, because they usually are handled at our adult ED. Both patient groups are likely to be the most easy to triage, leaving in our study populations an overweight of those who may be among the most difficult to triage. The studies were performed at a single center in a relatively large PED at a university hospital in Norway, and clinical data of included patients were not compared with the entire PED population. All these factors may limit the generalizability of our findings. In Study 1 it may be a limitation that only 12 out of 19 nurses were included in Wave B and the evaluation of intrarater agreement. However, from our experience, we believe there may be a substantial turnover of nurses in hospitals (e.g., in relation to pregnancy and continuing education). Because in Study 2 we included those nurses who were available during a 9-month period, it may be claimed that too many nurses were inexperienced with the RETTS-p system, despite the fact that all received standardized training. On the other hand, one may argue that both conditions may rather be advantages than limitations, because RETTS-p was studied under realistic, everyday circumstances in the PED.

## Conclusion

The Scandinavian triage system RETTS-p for children was easily implemented in a large Norwegian PED, and we found a very high degree of agreement between nurses who used RETTS-p to prioritize children in patient case scenarios as well as in real-life triaging. RETTS-p may be a credible and robust triage system in PEDs, but it has not been validated yet.

## Additional file

Additional file 1:
**The 20 cases which was used in study 1.** (PDF 146 kb) Additional file 2:
**An example of an ESS (Emergency Signs and Symptoms).** (PPTX 108 kb)

## Abbreviations

ED: emergency department
ESS: emergency signs and symptoms
ICC: intraclass correlation coefficient
RETTS-p: rapid emergency triage and treatment system-pediatric
VP: vital parameter
