# Supplementary material for: A reliability study of the rapid emergency triage and treatment system for children
Source: Scand J Trauma Resusc Emerg Med. 2016 Feb 24;24:19. doi: 10.1186/s13049-016-0207-6 (PMC4766636; doi:10.1186/s13049-016-0207-6)
Supplement: Additional file 1: — The 20 cases which was used in study 1. (PDF 146 kb) [file 13049_2016_207_MOESM1_ESM.pdf]

Attachments

1) An example of an ESS algorithm: ESS no. 104: Respiratory difficulty (see PowerPoint page).

2) An example of an age adjusted VP algorithm: VP algorithm for 3 to 5 month old children.

3–5 Months				
	RED	ORANGE	YELLOW	GREEN
A	Obstructed airway	-	-	-
B	RR <20 or >75, SATO2 <93% with O2	RR <25 or >65, SATO2 <93% without O2	RR <30 or >55. SATO2: 93–94%	RR 30–55; SATO2 95–100%
C	Pulse rate <70 or >210	Pulse rate < 80 or >180	Pulse rate < 100 or >160	Pulse rate 100–160
D	GCS <11 Ongoing cramps	GCS 11–13	Tired/weak	Alert
E	-	<36ºC or >39ºC	-	36ºC–39ºC

3) Case scenarios used in Study 1.

Case 1:

Boy 3.5 years: After kindergarten, at home he turned blue in his face, secretions around his mouth, and had a respiratory arrest for some seconds. No convulsions. Status on admission PED: Lethargic but with good contact. RR 26/min, SAT 100%, HR 152/min, capillary refill time 2 seconds, GCS 15, temperature 38.3°C rectally.

Case 2:

Boy 8 months: After dinner, sudden rash on face, back, and stomach. Swollen lips and face. Respiratory stridor. Vomiting. Went to the primary care ED, got steroids before arrival at the PED. Status on admission PED: Still rash, swollen face, and warm skin. Lethargic, good contact. Normal respiration. RR 36/min, SAT 99%, pulse 157/min, temperature 37.9°C rectally.

Case 3:

Girl 11 years: In bad condition last days. Today, in the morning, collapsed in the bathroom. Her mother says she was unconscious 20 seconds. No convulsions. Status on admission PED: Awake, good contact, pale. RR 20/min, SAT 100%, pulse 98/min, temperature 37.2°C temporalis, GCS 15.

Case 4:

Girl 14 years: Took unknown dose of paracetamol and ibuprofen at the same time. Tired in the afternoon and slept a lot. Vomiting at home. Arrived at the PED 8 hours after the tablet intake. Status on admission PED: Lethargic, good contact. Nauseous, vomiting. Cold skin peripherally. RR 22/min, SAT 99%, pulse 94, BT 123/74 (81), temperature 37.3°C temporalis.

Case 5:

Boy 2.5 years: Fever for 5 days starting with a “fevercramp.” Today, exacerbation with body pain and pain in the teeth. Red on left cheek. Status on admission PED: Awake, good contact. Grunting, cough. Pale, warm skin. RR°C 36/min, SAT 97%, pulse 150/min, temperature 38.9°C rectally.

Case 6:

Boy 1.5 years: Viral RS infection 1 week ago. Then better for some days, but last 2 days exacerbation with fever, cough, and respiratory distress. Little food and drink and less wet diapers. Status on admission PED: Expiratory stridor, sub- and intercostal retractions. Loose cough. RR 48/min, SAT 98%, pulse 178/min (crying), capillary response time 2 seconds, temperature 37.3°C rectally.

Case 7:

Boy 2.5 years: Known asthma. 2 days cold. Increased respiratory distress. Medicated with terbutaline and fluticason inhalations at home, no effect. Loose cough and stuffy nose. Status on admission PED: Awake, good contact. Grunting, jugular and intercostal retarctions. RR 22/min, SAT 94%, pulse 150/min, temperature 37.9°C temporalis.

Case 8:

Boy 8 years: Sudden onset headache, dizziness, vomiting. Corresponding symptoms months ago, improved spontaneusly. Status on admission PED: No headache, no dizziness, good contact and in good condition. RR 18/min, SAT 100%, pulse 87/min, BP 126/76 (86), GCS 15, temperature 36.8°C temporalis, VAS pain scale 0.

Case 9:

Girl 3.5 weeks: Sick for 4 days with respiratory tract infection. Yesterday she was more lethargic, little food yesterday, less wet diapers. Status on admission PED: Pale with cyanotic lips, loose cough, sub- and intercostal and jugular retractions, nasal flaring, expiratory stridor. RR 60/min, SAT 90%, pulse 200/min, capillary refill time 3 seconds, temperature 36.5°C rectally.

Case 10:

Boy 9 years: Today fell on the ice outside, hit his forehead, not unconscious. Was at the primary care ED, no treatment and went home. Later on the same day, growing sleepy, vomiting. Status on admission PED: Awake, good contact, and oriented. Nauseous, headache, no pain in his neck. RR 24/min, SAT 99%, pulse 104/min, BP 115/67 (78), GCS 15, normal pupils with normal light reaction, temperature 36.8°C temporalis. VAS pain scale 3.

Case 11:

Girl 2.5 years: Frequent diarrhea 1 week ago, vomiting 2 days after. Little food and drink. Last 2 days no diarrhea and vomiting, but lethargic and with fever. Little urine. Interval abdominal pain. Status on admission PED: Lethargic. RR 26/min, SAT 98%, pulse 142/min, capillary refill time 2 seconds, temperature 38.6°C rectally.

Case 12:

Girl 4 weeks: Recently had a RS viral infection. Today, stuffy nose and increased respiratory disease. Treatment at home with nasal drops without effect. Status on admission PED: Mild inter- and subcostal retractions. Awake and good contact. RR 52/min, SAT 95%, pulse 178/min, temperature 37.5°C rectally.

Case 13:

Girl 15 years: 2 days with cough, lot of secretions. Temporal pain, cold sweat, chills. Abdominal pain right side. Not nauseous. Status on admission PED: Awake, good contact, VAS pain scale 6, RR 14/min, SAT 98%, pulse 94/min, BP 104/69 (77), temperature 39.2°C temporalis.

Case 14:
Boy 10 months: Fever for 5 days, otitis, cough, and respiratory distress. Bad sleep. Vomiting breast milk. Status on admission PED: Awake, good contact. Wheezing respiration, expiratory prolongation. Sub- and intercostal retractions. RR 52/min, SAT 100%, pulse 135/min, temperature 36.6°C rectally.

Case 15:
Boy 6 years: Pain right hip/groin last 3 days. No trauma. Trouble standing on right leg. Constant pain. Fever last week, no fever at the moment. Status on admission PED: RR 22/min, SAT 100%, pulse 86/min, temperature 37.2°C temporalis, VAS pain scale 7.

Case 16:
Boy 7 months: Fever and lethargic last 5 days, questions about RS infection, cough, a lot of secretions. Vomiting after meal. Wet diapers. Status on admission PED: Tired, lethargic. Loose cough. RR 40/min, SAT 99%, pulse 120/min, temperature 37.0 rectally.

Case 17:
Girl 4 months: In bad condition yesterday, vomiting, loose stools. Less drink, vomiting after breastfeeding. Less urine in diapers. Status on admission PED: In good condition, smiling. RR 42/min, SAT 100%, pulse 159/min, capillary refill time 2 seconds, temperature 37.1°C rectally.

Case 18:
Boy 15 years: Bad shape and tired last month. Lot of drinking, frequent urine, especially at night. Less to eat, weight loss 5 kg. Last 2 days abdominal pain and vomiting. Loose stools. Status on admission PED: Lethargic. Thirsty. Abdominal pain, vomiting. RR 26/min, SAT 97%, pulse 122/min, temperature 37.1°C, VAS pain scale 6, blood sugar level 22 mmol/l.

Case 19:
Girl 8 years: 14 days ago suspected appendicitis. Today again powerful abdominal pain. Paracetamol without effect. CRP and urine stix negative at the primary care ED. Status on admission PED: Awake. Right side abdominal pain, tender to palpation. RR 22/min, SAT 100%, pulse 69/min, temperature 37.2°C temporalis, VAS pain scale 7.

Case 20:

Girl 6 years: Sick for 5 days, fever 40.0. Pain in ears and the head. CRP 128 mg/L at the primary care ED last day. Little food and drink. Less dark urine. Little sleep. Pain left ear. Yesterday vomiting, but not today. CRP 187 mg/L at the general practitioner. Status on admission to PED: Pale and marbled. Lethargic and irritable. RR 23/min, SAT 100%, pulse 120/min, temperature 37.6°C temporalis, BP 101/61 (69), capillary refill time 2 seconds.
