# Supplementary material for: A reliability study of the rapid emergency triage and treatment system for children
Source: Scand J Trauma Resusc Emerg Med. 2016 Feb 24;24:19. doi: 10.1186/s13049-016-0207-6 (PMC4766636; doi:10.1186/s13049-016-0207-6)
Supplement: Additional file 2: — An example of an ESS (Emergency Signs and Symptoms). (PPTX 108 kb) [file 13049_2016_207_MOESM2_ESM.pptx]

## Slide 1
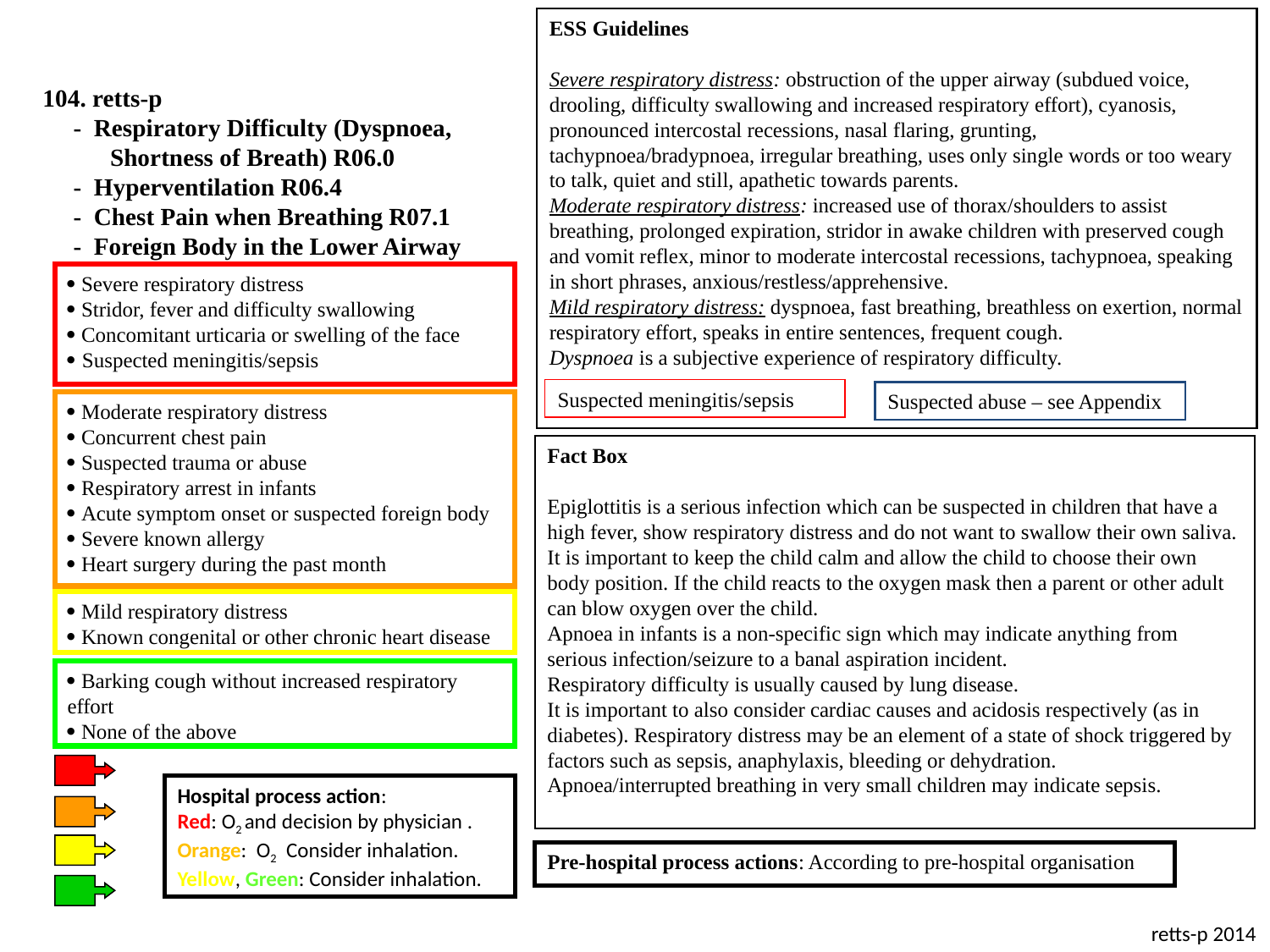

ESS Guidelines
Severe respiratory distress: obstruction of the upper airway (subdued voice, drooling, difficulty swallowing and increased respiratory effort), cyanosis, pronounced intercostal recessions, nasal flaring, grunting, tachypnoea/bradypnoea, irregular breathing, uses only single words or too weary to talk, quiet and still, apathetic towards parents.
Moderate respiratory distress: increased use of thorax/shoulders to assist breathing, prolonged expiration, stridor in awake children with preserved cough and vomit reflex, minor to moderate intercostal recessions, tachypnoea, speaking in short phrases, anxious/restless/apprehensive.
Mild respiratory distress: dyspnoea, fast breathing, breathless on exertion, normal respiratory effort, speaks in entire sentences, frequent cough.
Dyspnoea is a subjective experience of respiratory difficulty.
Suspected meningitis/sepsis
Suspected abuse – see Appendix
104. retts-p
 - Respiratory Difficulty (Dyspnoea,
 Shortness of Breath) R06.0
 - Hyperventilation R06.4
 - Chest Pain when Breathing R07.1
 - Foreign Body in the Lower Airway
 - Apnoea/Interrupted Breathing
 Severe respiratory distress
 Stridor, fever and difficulty swallowing
 Concomitant urticaria or swelling of the face
 Suspected meningitis/sepsis
 Moderate respiratory distress
 Concurrent chest pain
 Suspected trauma or abuse
 Respiratory arrest in infants
 Acute symptom onset or suspected foreign body
 Severe known allergy
 Heart surgery during the past month
Fact Box
Epiglottitis is a serious infection which can be suspected in children that have a high fever, show respiratory distress and do not want to swallow their own saliva.
It is important to keep the child calm and allow the child to choose their own body position. If the child reacts to the oxygen mask then a parent or other adult can blow oxygen over the child.
Apnoea in infants is a non-specific sign which may indicate anything from serious infection/seizure to a banal aspiration incident.
Respiratory difficulty is usually caused by lung disease.
It is important to also consider cardiac causes and acidosis respectively (as in diabetes). Respiratory distress may be an element of a state of shock triggered by factors such as sepsis, anaphylaxis, bleeding or dehydration.
Apnoea/interrupted breathing in very small children may indicate sepsis.
 Mild respiratory distress
 Known congenital or other chronic heart disease
 Barking cough without increased respiratory effort
 None of the above
Hospital process action:
Red: O2 and decision by physician .
Orange: O2 Consider inhalation. Yellow, Green: Consider inhalation.
Pre-hospital process actions: According to pre-hospital organisation
retts-p 2014
